# Analysis of Pigmentation Changes in Bracts of *Bougainvillea* × *buttiana* ‘Miss Manila’ During Different Developmental Periods

**DOI:** 10.3390/biology14111607

**Published:** 2025-11-17

**Authors:** Xiangdong Liu, Yuwan Ma, Jiawen Yan, Yan Liu, Yaqi Huang, Siyin Deng, Jiawen Dong, Yulin Hu

**Affiliations:** 1 College of Agricultural and Forestry Sciences, Hunan Applied Technology University, Changde 415000, China; m15116158531_1@163.com (X.L.); 2 Hunan Botanical Garden, Changsha 410116, China; 3 Hunan Changsha-Zhuzhou-Xiangtan City Cluster Ecosystem Observation and Research Station, Changsha 410116, China; 4 College of Agronomy, Hunan Agricultural University, Changsha 410128, China; mayuwan2021@163.com; 5 Changde Engineering Technology Research Center for Ecological Process Regulation and High-Value Utilization of Economic Forests, Changde 415000, China

**Keywords:** bract development, coloring, coloration, expression analysis

## Abstract

**Simple Summary:**

To investigate pigmentation changes and the expression of pigment synthesis-related genes in the bracts of *Bougainvillea* × *buttiana* ‘Miss Manila’ at different developmental stages, we measured color parameters, pigment contents, and gene expression levels. The results showed that the bracts developed through four distinct stages. Color saturation, brightness, and redness increased throughout development. Chlorophyll content peaked at the bract stage (2.2 mg/g), while flavonoids and betalains began to accumulate from the young stage, with betacyanin reaching its highest level at full bloom (4.94 mg/g). Chlorophyll a, betacyanin, and flavonoids showed significant correlations with color changes. Genes involved in the betalain, flavonoid, and chlorophyll metabolic pathways showed distinct expression patterns. In conclusion, the coloration of the bracts is co-regulated by these three metabolic processes, with key genes playing crucial roles. These findings provide a theoretical foundation for the landscape cultivation and flower color improvement of *Bougainvillea*.

**Abstract:**

*Bougainvillea* has large and vibrant-colored bracts, which are widely used in landscape gardening. In order to study the changing pattern of pigmentation and the expression of genes related to pigment synthesis during different developmental periods of the bracts of *B. × buttiana*, we determined the color parameters of the bracts of *B. × buttiana* has by using colorimetric color cards and colorimeters, and quantitatively determined the contents of chlorophyll a, chlorophyll b, betacyanin, betaxanthin and flavonoids, and the expression of genes related to pigment synthesis was detected during the BR1 and BR3. The results showed that the bracts of *B. × buttiana* ‘Miss Manila’ can be classified into four distinct growth and developmental stages, namely Bract Primordial Stage (BR1), Bract Color Transition Stage (BR2), Bract Maturation Stage (BR3), and Bract Senescence Stage (BR4). The BR1 have lower color saturation, brightness, and redness, and with bract development, their color saturation, brightness, and redness gradually increased. In addition, chlorophyll content was highest at the BR1 (2.2 mg/g), and from the BR1, flavonoids and betalain content began to increase, and higher betacyanin content in all stages, with betacyanin being the highest at the BR3 at 4.94 mg/g. Correlation analysis of the color parameters with pigment content showed that chlorophyll a, betacyanin, and flavonoid contents were significantly correlated with the bract color changes. With bract development, the betalain metabolism pathway *BgDODA* gene was significantly up-regulated; the flavonoid metabolism pathway *BgDFRA* and *BgF3H* genes were significantly up-regulated, whereas the *BgDTX*, *BgFLS*, and *BgCHIL* genes were significantly down-regulated; and the chlorophyll metabolism pathway *BgSGR* gene was significantly up-regulated, whereas the *BgPORA* gene was down-regulated in expression. ProtParam-based analysis characterized the *BgFLS*-encoded protein as a stable, hydrophilic 2-oxoglutarate-dependent oxidoreductase lacking transmembrane domains and a signal peptide, and the *BgCHIL*-encoded protein as a stable, hydrophilic chalcone isomerase also lacking transmembrane domains and a signal peptide. In summary, betalain metabolism, flavonoid metabolism and chlorophyll metabolism jointly regulate the bract color change of *B. × buttiana* has, and it is possible that the genes of *BgCHIL*, *BgFLS*, *BgSGR*, and *BgF3H* are involved in the regulation of the bract color change of *B. × buttiana*.

## 1. Introduction

*Bougainvillea* × *buttiana* is an evergreen vine-like climbing shrub belonging to the genus *Bougainvillea* within the family Nyctaginaceae [1]. Its primary ornamental feature is the color of bracts. The cultivar *B. × buttiana* has red bracts with vibrant coloration and exhibits relatively high cold tolerance, making it a popular variety in the market [2].

The pigments responsible for bract coloration in *Bougainvillea* are generally considered to form similarly to those in the leaves of variegated plants. Plant pigments constitute the material basis for flower and leaf coloration and primarily include chlorophylls, carotenoids, flavonoids, and betalains [3]. Among these, carotenoids, flavonoids, and chlorophyll exist in the majority of plants in nature, whereas betalains are present in about 17 plant families of the order Caryophyllales [4]. They mainly include betacyanins (red-violet pigments) and betaxanthins (yellow-orange pigments) [5]. Plant pigments have potential applications in fields such as food, medicine, and cosmetics. Beyond imparting color to flowers, fruits, and leaves to attract insects and enhancing plant stress resistance, betalains also exhibit excellent antioxidant properties [6]. Simultaneously, flavonoids possess various biological activities, including antioxidant, antibacterial, and antiviral effects [2]. Research shows that certain *Bougainvillea* varieties contain very high levels of betalain pigments. Therefore, *Bougainvillea* is an excellent source for extracting natural pigments [7].

The pigment composition in *Bougainvillea* cultivars primarily includes carotenoids, chlorophyll, flavonoids [8], and betalains. Notably, *Bougainvillea* lacks anthocyanins, which is consistent with the fact that betalains and anthocyanins are mutually exclusive pigments [9]. Previous studies suggest that the coloration of *Bougainvillea* bracts is primarily achieved through the betalain biosynthetic pathway [10]. The betalain biosynthetic pathway is mainly catalyzed and regulated by the enzyme 4, 5-DOPA dioxygenase (DOD) [11]. The betalain biosynthetic pathway, flavonoid biosynthetic pathway, phenylpropanoid pathway, and carotenoid biosynthetic pathway collectively regulate the content of flavonoids and betalains within *Bougainvillea* bracts, thereby modulating bract coloration [12,13].

The biosynthesis and accumulation of these pigments are tightly regulated by key structural and regulatory genes within their respective pathways. For instance, in the flavonoid pathway, genes encoding enzymes such as chalcone synthase (CHS), chalcone isomerase (CHIL), flavanone 3-hydroxylase (F3H), flavonol synthase (FLS), and dihydroflavonol 4-reductase (DFR) are fundamental for the production of various flavonoid compounds [14]. Similarly, chlorophyll metabolism is governed by genes involved in its synthesis (e.g., protochlorophyllide oxidoreductase, POR) and degradation (e.g., STAY-GREEN, SGR) [15]. Therefore, a comprehensive analysis of these gene families, alongside the betalain-related gene DOD, is crucial for a holistic understanding of the color formation in Bougainvillea bracts. However, the expression patterns and roles of these key chlorophyll and flavonoid metabolism genes during the bract development of *Bougainvillea* remain largely unexplored.

Currently, research on the color parameters, pigment content and composition, and their interrelationships in the bracts of *B. × buttiana* has at different developmental stages is limited. This study aimed to investigate the pigment composition and coloration mechanisms in the bracts of *B. × buttiana* has at various developmental stages by comparatively analyzing bract pigment content, the expression levels of genes involved in pigment biosynthesis, and conducting bioinformatic analysis of the proteins encoded by key genes. The findings provide a reference for exploiting new pigment resources and enhancing the economic utilization value of *B. × buttiana*.

## 2. Materials and Methods

### 2.1. Plant Material

The experiments were conducted on *B. × buttiana* ‘Miss Manila’. The plants were grown at the Flower Cultivation Base of Hunan Agricultural University. Plants were potted in 25 cm diameter plastic pots filled with a peat moss:perlite:garden soil (2:1:1, *v*/*v*/*v*) mixture. During growth, they were watered regularly to maintain soil moisture and fertilized weekly with a balanced compound fertilizer (N-P-K: 20-20-20). All plants were maintained under full sunlight in an open field. Uniform, healthy, and disease-free individuals were selected as experimental materials. On sunny days between 9:00 and 10:00 AM, well-developed sun-exposed branches were chosen. Bracts at different developmental stages from the upper parts of these branches were collected, immediately placed in ice-cooled containers, and transported to the laboratory for subsequent measurement and analysis. Three biological replicates were performed.

### 2.2. Measurement of Color Parameters

**Figure 1:**
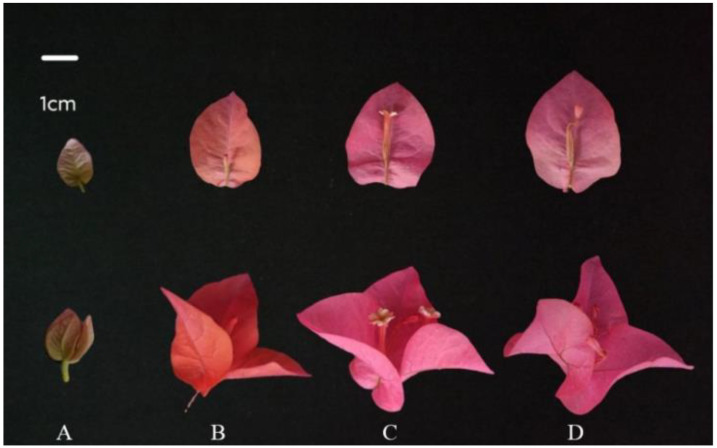
Phenotypes of bracts and perianth tube at different stages of development. (**A**) Bract Primordial Stage (BR1); (**B**) Bract Color Transition Stage (BR2); (**C**) Bract Maturation Stage (BR3); (**D**) Bract Senescence Stage (BR4). The upper row illustrates the morphological variations of the perianth tube across various developmental stages (Each bract subtends a single flower), while the lower row displays the morphology of complete inflorescence with primary emphasis on bract morphology.

Colorimetric analysis was performed using a chroma meter (3 nh Spectrophotometer, model YS3020, Guang zhou, China) under diffuse indoor lighting conditions to avoid direct sunlight. For each bract, the measurement point was located at the petiole base. Bracts of *B. × buttiana* ‘Miss Manila’ at different developmental stages (Bract Primordial Stage (BR1), Bract Color Transition Stage (BR2), Bract Maturation Stage (BR3), and Bract Senescence Stage (BR4). were measured (Figure 1). Lightness (L*), red–green component (a*), and blue–yellow component (b*) values were recorded directly by the instrument. Chroma (C) was calculated using the formula: C = (a*^2^ + b*^2^)^1/2^. Three measurements were taken per bract (technical replicates).

### 2.3. Chlorophyll Extraction and Content Determination

Chlorophyll content was extracted and determined using a 96% (*v*/*v*) ethanol immersion method [16]. Collected bracts of *B. × buttiana* ‘Miss Manila’ were cut into thin strips, and major veins and petioles were removed. Approximately 0.1 g of bract tissue from each developmental stage was weighed using a PB303-E electronic balance (Mettler Toledo, Greifensee, Switzerland). The weighed tissue was placed into a 10 mL centrifuge tube and immersed in 96% (*v*/*v*) ethanol. Tubes were capped tightly, shaken periodically during the extraction process, and kept in the dark until the strips turned completely white. The tissue strips were then removed, and the volume in each tube was adjusted to 10 mL with 96% (*v*/*v*) ethanol. The resulting chlorophyll extract was transferred into a 1 cm pathlength cuvette. Absorbance (A) was measured at wavelengths of 665 nm and 649 nm using a spectrophotometer (UV-1800, Shimadzu, Tokyo, Japan), with 96% (*v*/*v*) ethanol serving as the blank reference. Chlorophyll concentration was calculated according to the Beer–Lambert law. Three biological replicates were performed for each developmental stage. Chlorophyll a and b contents were calculated according to the Arnon formula [17].

### 2.4. Flavonoid Extraction and Content Determination

Flavonoid extraction and quantification were performed according to the method described by Zhang et al. [8]. Collected bracts of *B. × buttiana* ‘Miss Manila’ at the four developmental stages (BR1, BR2, BR3, BR4) were cut into thin strips using clean scissors, and major veins and petioles were removed. One milliliter of 60% ethanol was added to the sample, followed by ultrasonic extraction at 60 °C for 30 min. The mixture was then centrifuged at 12,000 r·min^−1^ for 10 min, and the supernatant was collected. The supernatant was diluted to a final volume of 1 mL with ethanol solution of the same concentration to obtain the test sample solution. The total flavonoid content was determined using the sodium nitrite-aluminum nitrate colorimetric method. Absorbance was measured at 470 nm using a spectrophotometer (UV-1800, Shimadzu, Tokyo, Japan), with distilled water serving as the blank reference. Three biological replicates were conducted for each developmental stage.

### 2.5. Betalain Extraction and Content Determination

Betalain extraction and quantification were conducted following the method described by Zhang et al. [8]. Collected bracts of *B. × buttiana* ‘Miss Manila’ at the four developmental stages (BR1, BR2, BR3, BR4). Bracts from different developmental stages (0.1 g each) were weighed using a PB303-E electronic balance, thoroughly ground, and added to 1.5 mL of methanol pre-cooled to 4 °C. The mixture was immediately vortexed vigorously and extracted at 4 °C for 1 h. Absorbance values for betacyanins were measured at 538 nm, and for betaxanthins at 465 nm, using a UV spectrophotometer (UV-1800, Shimadzu, Tokyo, Japan). Three biological replicates were performed for each developmental stage. Betalain content was calculated using the following formula:

Betalain content (mg/g) = [BC (mg/L) × V (mL)]/[FW (g) × 1000]

BC (mg/L) = (OD × DF × MW × 1000)/(ε × L)

Parameters:

OD (Optical Density): Absorbance at 538 nm and 465 nm; DF: Dilution factor (dimensionless); MW: Molecular weight (betacyanin = 550 g/mol; betaxanthin = 308 g/mol); ε: Molar extinction coefficient (betacyanin = 6 × 10^4^ L·mol^−1^·cm^−1^; betaxanthin = 4.8 × 10^4^ L·mol^−1^·cm^−1^); L: Cuvette pathlength (cm); V: Total extraction volume (mL); FW: Fresh weight of sample (g).

### 2.6. Gene Expression Quantification

Total RNA was isolated from different tissues using the FastPure Universal Plant Total RNA Isolation Kit (Vazyme Biotech Co., Ltd., China) according to the manufacturer’s instructions. After assessing RNA purity and quality, cDNA was synthesized using the HiScript II Q RT SuperMix for qPCR kit (Vazyme Biotech Co., Ltd., China). Real-time quantitative PCR (RT-qPCR) was performed using the ChamQ Universal SYBR qPCR Master Mix (Vazyme Biotech Co., Ltd., China), with both the reaction setup and protocol strictly following the manufacturer’s recommendations. Nine key genes associated with color changes were selected and their expression was validated at the BR1 and BR3 stages: *BgDODA*, *BgDFRA*, *BgF3H*, *BgSGR*, *BgPORA*, *BgDTX*, *BgFLS*, *BgCHIL*, and *BgCHS*. Bract samples at the BR1 and BR3 stages were rapidly transferred to a high-speed tissue grinder pre-cooled with liquid nitrogen for thorough homogenization. Total RNA from the samples was extracted using the FastPure Universal Plant Total RNA Isolation Kit (Vazyme Biotech Co., Ltd., Nanjing, China). RNA samples stored at −80 °C were retrieved and placed on a clean bench; all subsequent operations were performed on ice to prevent RNA degradation. Components were added to PCR tubes according to the table below to prepare the RNA template solution. The prepared solution was then transferred to a PCR instrument and subjected to denaturation and annealing reactions following the pre-set program (Table 1).

The solution was gently mixed using a pipette, centrifuged with a portable centrifuge, and then placed in a PCR thermocycler for denaturation and annealing. The reaction program was set as follows: 70 °C for 3 min, followed by holding at 4 °C for later use.

Subsequently, the following components were added to the aforementioned reaction solution to prepare a reverse transcription reaction system, and cDNA was synthesized (Table 2).

The aforementioned reaction solution was gently mixed using a pipette, centrifuged with a portable centrifuge, and then placed in a PCR instrument for the reaction. The reaction program was set as follows: 42 °C for 60 min, 70 °C for 15 min, followed by holding at 4 °C. After the reaction, the solution was stored at −20 °C for later use.

Gene-specific primers were designed using Primer Premier 5.0 software. The measurement was performed using a Bio-Rad (Hercules, CA, USA) instrument. qPCR reaction conditions are shown in Table 3.The ACTIN gene was used as the internal reference control. Three biological replicates were performed for each experiment. The relative gene expression levels were calculated using the 2^–ΔΔCt^ method. The sequences of the primers used are listed in Table 4. All primers were synthesized by Tsingke Biotechnology Co., Ltd. (Beijing, China).

### 2.7. Sequence Bioinformatics Analysis

ProtParam (http://web.expasy.org/protparam/, accessed on 8 July 2025) was used to predict the physicochemical properties of the protein, including molecular weight and isoelectric point; TMHMM 2.0 (https://services.healthtech.dtu.dk/services/TMHMM-2.0/, accessed on 8 July 2025) was employed to analyze the transmembrane structure of the protein; SignalP 4.1 Server (https://services.healthtech.dtu.dk/services/SignalP-4.1/, accessed on 8 July 2025) was utilized to analyze the protein’s signal peptide; SOPMA (https://npsa-prabi.ibcp.fr/cgi-bin/npsa_automat.pl?page=npsa%20sopma.html, accessed on 8 July 2025) was applied to analyze the protein’s secondary structure; and SWISS-MODEL (https://swissmodel.expasy.org/, accessed on 8 July 2025) was used to analyze the protein’s tertiary structure.

### 2.8. Data Analysis

Experimental data were entered using Microsoft Excel 2011 software. Subsequently, the experimental results were visualized using GraphPad Prism 9 software. A one-way analysis of variance (ANOVA) was performed on the mean of the experimental data using SPSS (ver. 25.0) for Windows (SPSS Inc., Chicago, IL, USA) with the honestly significant difference test of LSD, Duncan’s test, and Dunnett’s test.

## 3. Results

### 3.1. Determination of Developmental Stages in B. × buttiana ‘Miss Manila’ Bracts

Based on the growth and development process, the bracts were divided into four distinct stages: (1) BR1 (bracts green with a slight reddish tinge, no flowers present); (2) BR2 (bracts reddish-orange, flower buds formed but not open); (3) BR3 (bracts rose-red, inflorescence partially or fully open); (4) BR4 (bracts rose-red, flowers partially or completely withered) (Figure 1).

### 3.2. Color Parameters of B. × buttiana ‘Miss Manila’ Bracts at Different Developmental Stages

The bract color of *B. × buttiana* ‘Miss Manila’ was light green with a slight reddish tinge at BR1, transitioned to reddish-orange at BR2, and finally developed into rose-red at BR3 and BR4 (Table 5). Significant differences and discernible trends in bract color parameters were observed across the developmental stages (*p* < 0.05). As the bracts developed, the lightness (L*), chroma (C*), and red–green component (a*) values exhibited a progressive increase, reaching their maxima at the BR4. Conversely, the blue–yellow component (b*) values were consistently negative throughout all stages (Table 5).

### 3.3. Pigment Content in B. × buttiana ‘Miss Manila’ Bracts at Different Developmental Stages

At the initial developmental stage (BR1), chlorophyll was the predominant pigment, with chlorophyll a at 1.23 mg/g and chlorophyll b at 0.97 mg/g (Table 6). As bract development progressed, chlorophyll gradually degraded, while the contents of flavonoids and total betalains began to increase at BR2. During the BR2, BR3, and BR4, betacyanins were the primary pigments, with contents of 4.45 mg/g, 4.94 mg/g, and 4.69 mg/g, respectively. Flavonoids and betaxanthins were present at lower levels during these stages (Table 6).

### 3.4. Correlation Between Color Parameters and Pigment Content in Bracts of a B. × buttiana ‘Miss Manila’ at Different Developmental Stages

**Figure 2:**
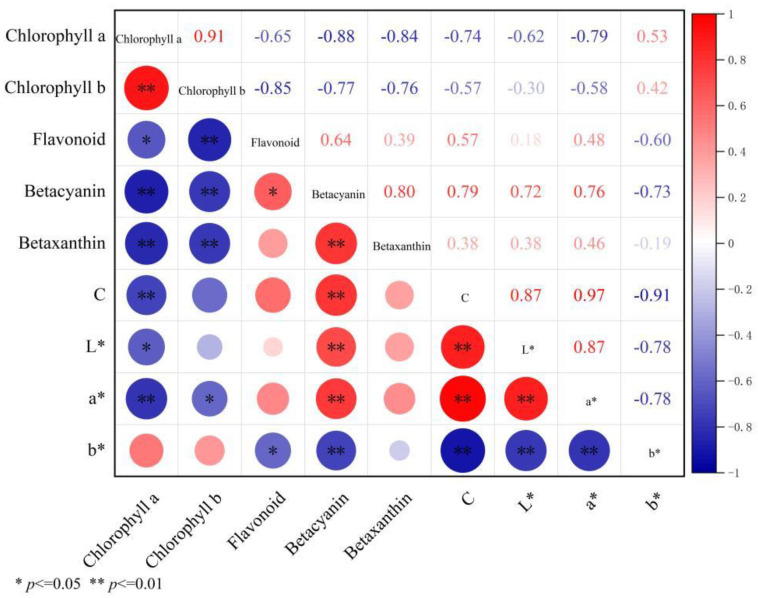
Correlation between color parameters and pigment content of bracts at different developmental periods. * (*p* ≤ 0.05); ** (*p* ≤ 0.01).

To investigate the relationship between bract color parameters and pigment content, correlation analysis was performed. As shown in Figure 2, the bract color parameter C* showed a highly significant negative correlation with chlorophyll a (r = −0.74, *p* < 0.01) and a negative correlation with chlorophyll b, though not significant. C* exhibited a highly significant positive correlation with betacyanin (r = 0.79, *p* < 0.01) and positive correlations with flavonoids and betaxanthin. L* had a significant negative correlation with chlorophyll a (r = −0.62, *p* < 0.05) and a highly significant positive correlation with betacyanin (r = 0.72, *p* < 0.01). The a* showed significant negative correlations with chlorophyll a (r = −0.79, *p* < 0.01) and chlorophyll b (r = −0.58, *p* < 0.05), and a* highly significant positive correlation with betacyanin (r = 0.76, *p* < 0.01). The b* exhibited negative correlations with chlorophyll a and chlorophyll b, but these were not significant. However, b* showed significant negative correlations with flavonoids (r = −0.60, *p* < 0.05) and betacyanin (r = −0.73, *p* < 0.01).

To elucidate the relationship between bract color parameters and pigment content in *B. × buttiana* ‘Miss Manila’, multiple linear regression analysis was performed (Table 7). Chroma C, L*, a*, and b* were set as dependent variables, while the content of chlorophyll a, chlorophyll b, flavonoid, betacyanin, and betaxanthin were used as independent variables. Based on the absolute values of the coefficients in the fitted equations, the influence of bract pigment content on different color parameters varied. According to the standardized regression coefficients (Beta), chlorophyll b exhibited the strongest influence on the color parameters Chroma C, L*, and a*, while flavonoid content showed the strongest effect on b* (Table 7).

### 3.5. Expression Patterns of Pigment Biosynthesis-Related Genes in Bracts of B. × buttiana ‘Miss Manila’ at Different Developmental Stages

**Figure 3:**
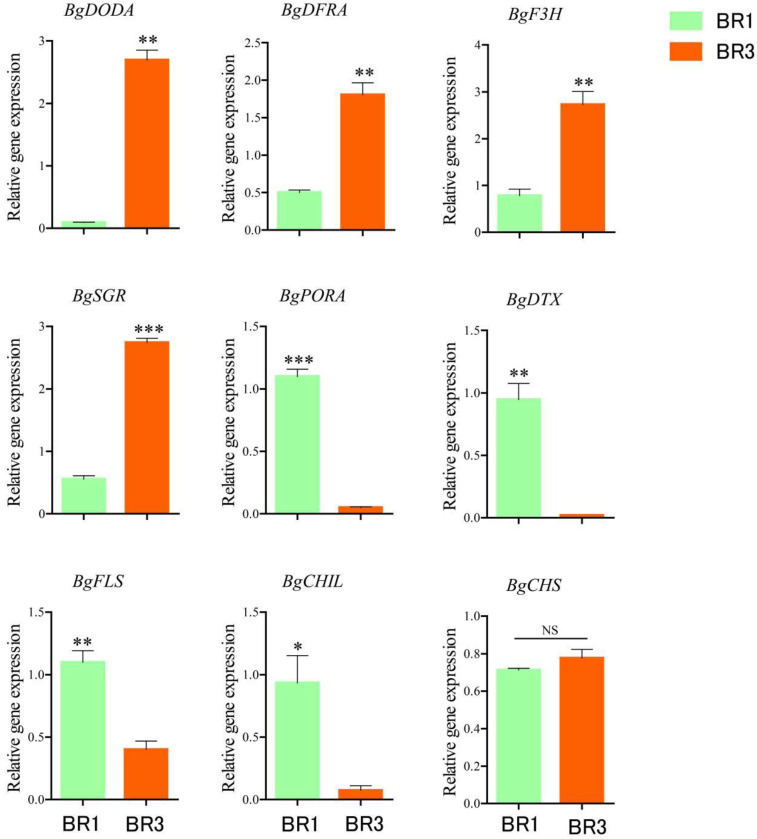
Expression patterns of pigment synthesis-related genes in bracts at different developmental periods. Data are presented as mean ± SD (n = 3). Error bars represent standard deviation (SD). * (*p* < 0.05) ** (*p* < 0.01) *** (*p* < 0.001).

In the betalain biosynthesis pathway, the 4,5-DOPA dioxygenase gene *BgDODA* was up-regulated at the BR3 compared to the BR1 (Figure 3). Moreover, *BgDODA* exhibited the highest level of up-regulation among the genes examined.

In the flavonoid biosynthesis pathway, the dihydroflavonol 4-reductase gene *BgDFRA* and the flavanone 3-β-hydroxylase gene *BgF3H* were up-regulated at the BR3 relative to the BR1. Conversely, the flavonol synthase gene *BgFLS* was down-regulated. The chalcone synthase gene *BgCHS* showed no significant difference in expression level between the two stages.

In the chlorophyll synthesis and metabolism pathway, the protochlorophyllide oxidoreductase gene *BgPORA* was down-regulated, while the chlorophyll degradation-related enzyme gene STAY-GREEN *BgSGR* was up-regulated at the BR3 compared to the BR1, with *BgSGR* showing a relatively high level of up-regulation.

These results indicate that the *BgDODA* gene in the betalain biosynthesis pathway, the *BgDFRA* and *BgF3H* genes in the flavonoid biosynthesis pathway, and the *BgSGR* gene in the chlorophyll metabolism pathway may play key regulatory roles in bract color change in *B. × buttiana* ‘Miss Manila’.

### 3.6. Physicochemical Properties and Structural Prediction of BgFLS and BgCHIL

**Figure 4:**
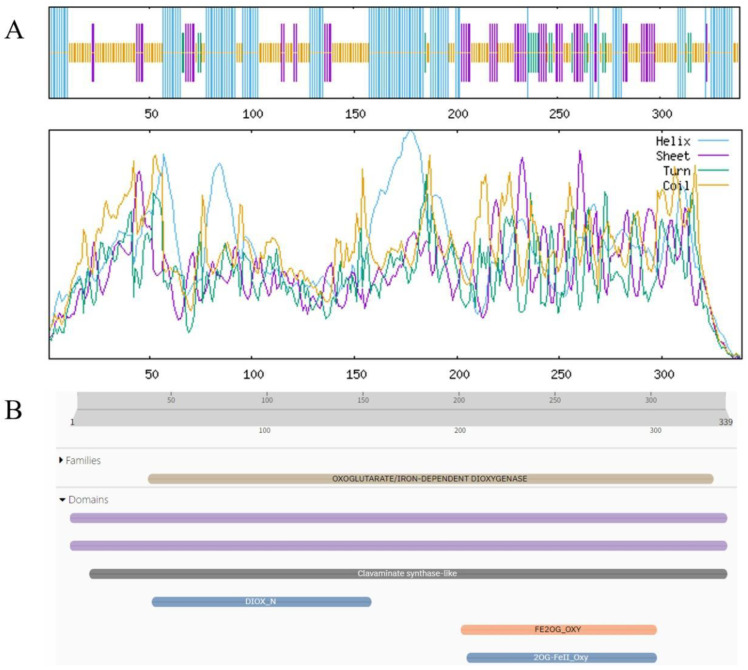
Physicochemical Properties and Structural Prediction of BgFLS. (**A**) BgFLS secondary structure prediction, (**B**) BgFLS conserved structural domains.

Analysis results of ProtParam and Structural Prediction of BgFLS-encoded Oprotein results from ProtParam analysis indicated that the molecular formula of the protein encoded by BgFLS is C_1730_H_2697_N_457_O_501_S_8_, with a predicted molecular weight of 38170.69. Its theoretical isoelectric point (pI) is 6.12, containing 42 positively charged residues (Arg + Lys) and 46 negatively charged residues (Asp + Glu). The predicted half-life of the protein is 30 h, and its instability index is 36.03, indicating it is a stable protein. Additionally, its grand average of hydropathicity (GRAVY) is −0.439, classifying it as a hydrophilic protein. Transmembrane structure prediction of the protein was performed using the online tool TMHMM, and the results revealed the absence of transmembrane domains (Figures S1 and S2). Prediction via the online tool SignalP 4.0 Server showed a mean S-score of 0.45, which is less than 0.5, suggesting the protein lacks a signal peptide. Furthermore, secondary structure prediction analysis found that in the structure of the BgFLS-encoded protein, α-helix accounts for 33.04%, random coil for 43.66%, β-turn for 5.60%, and extended strand for 17.70% (Figure 4A). Conserved domain analysis of the sequence using Pfam demonstrated that BgFLS encodes 339 amino acids, belongs to the Plant 2-oxoglutarate-dependent oxidoreductases family, and contains a clavaminate synthase-like domain (Figure 4B). The predicted tertiary structure of the protein is shown in Figure (Figure S3).

**Figure 5:**
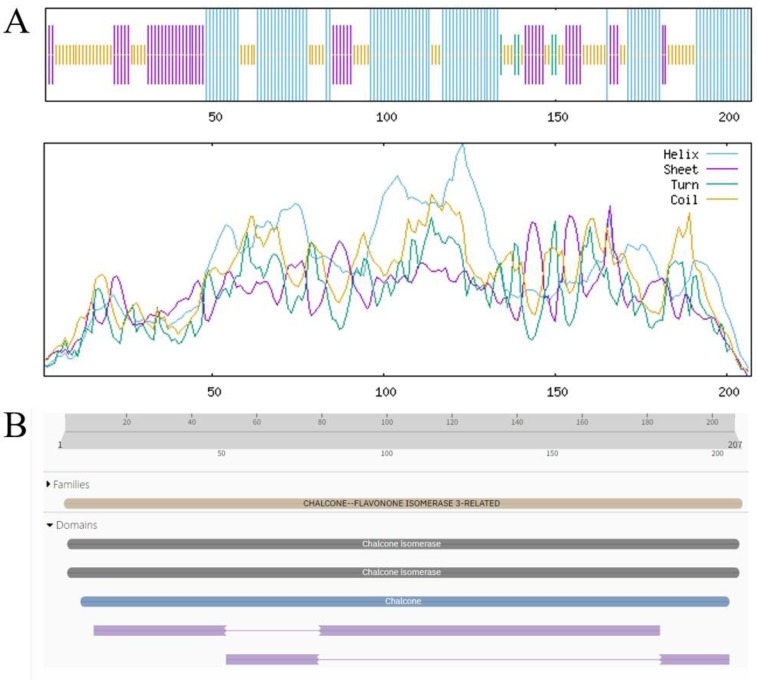
Physicochemical Properties and Structural Prediction of BgCHIL. (**A**) BgCHIL secondary structure prediction, (**B**) BgCHIL conserved structural domain.

Analysis Results of ProtParam and Structural Prediction of BgCHIL-encoded Protein Results from ProtParam analysis indicated that the molecular formula of the protein encoded by BgCHIL is C_1033_H_1610_N_258_O_315_S_5_, with a predicted molecular weight of 22,843.99. Its theoretical isoelectric point (pI) is 5.04, containing 19 positively charged residues (Arg + Lys) and 28 negatively charged residues (Asp + Glu). The predicted half-life of the protein is 30 h, and its instability index is 34.53, indicating it is a stable protein. Additionally, its grand average of hydropathicity (GRAVY) is −0.066, classifying it as a hydrophilic protein. Transmembrane structure prediction of the protein was performed using the online tool TMHMM, and the results revealed the absence of transmembrane domains (Figures S4 and S5). Prediction via the online tool SignalP 4.0 Server showed a mean S-score of 0.45, which is less than 0.5, suggesting the protein lacks a signal peptide. Secondary structure prediction analysis found that in the structure of the BgCHIL-encoded protein, α-helix accounts for 43.00%, random coil for 31.88%, β-turn for 2.42%, and extended strand for 22.71% (Figure 5A). For conserved domain analysis of the sequence using Pfam, it was found that BgCHIL encodes 207 amino acids, belongs to the CHALCONE–FLAVONONE ISOMERASE 3-RELATED family, and contains Chalcone isomerase and Chalcone-flavanone isomerase domains (Figure 5B). The predicted tertiary structure of the protein is shown in Figure S6.

## 4. Discussion

Correlations exist between plant color parameters and pigment types/contents, although the specific manifestations of these relationships vary among different plant species [18,19]. In the present study, similar results were observed: a significant positive correlation existed between L* and C, and between betacyanin content and the a* value during the development of *B. × buttiana* ‘Miss Manila’ bracts. When the betacyanin content in the bracts of *B*. *spectabilis* is relatively high, the red–green value a* is also relatively high [7]. Hierarchical cluster analysis and visual observations were used to divide *Lilium* cultivar group into five major color lines: white, pink, red, purple, and fuchsia, The brightness (L*) of the flower color gradually decreased with an increase in anthocyanin content, *Lilium* redness (a*) of the flower color was significantly negatively correlated with total anthocyanin (TA) content in the fuchsia line but was positively correlated with TA in the remaining four color lines [20]. In the present study, analogous results were observed: a significant positive correlation existed between L* and C and between betacyanin content and the a* value during the development of *B. × buttiana* ‘Miss Manila’ bracts. Furthermore, during bract development, the increase in flavonoid and betacyanin content, coupled with the decrease in chlorophyll content, leads to enhanced absorption of green light. This results in the bracts gradually turning red, a reduction in green purity, and mutual cancellation of red and green hues. Concurrently, increased absorption of yellow light occurs, causing a decrease in the b* value. In contrast, C, L*, and the a* value increase as the flowering stages progress. This signifies a progressive enhancement in bract color saturation, brightness, and redness, culminating in a highly saturated bright red appearance.

Bracts are modified leaves. During the color-changing process of *B. × buttiana* ‘Miss Manila’ bract development, chlorophyll content was the highest at the BR1 and was the BR2 and BR3. Conversely, the contents of flavonoids and betalains showed an opposite trend. According to the conclusions previously obtained on colored-leaf plants, the degree of leaf coloration is often determined by the ratio of flavonoids to chlorophyll [21,22,23]. The relative ratios of flavonoids to chlorophyll obtained in this study were calculated as follows: 0.85 at the BR1, 1.84 BR2, 3.58 at the BR3, and 3.41 at the BR4. As the ratio increased, the green color gradually faded while the red color became increasingly intense. A reciprocal relationship (where chlorophyll increases as the flavonoids and betalains decrease) was observed between chlorophyll, flavonoids and betalains, which is consistent with the conclusions previously drawn for color-leaved plants [24,25].

Bracts acquiring a more vibrant coloration are enabled to partially fulfill floral organ functions, such as attracting insect pollinators and protecting floral structures. The a* represents the degree of redness or greenness in color. In this study, the a* value was negatively correlated with chlorophyll a content and chlorophyll b content, but positively correlated with betacyanin content, betaxanthin content, and flavonoid content. This indicates that as the *B. × buttiana* ‘Miss Manila’ bracts develop, a higher a* value corresponds to redder coloration, lower chlorophyll content, and higher levels of betalains and flavonoids. Furthermore, multiple linear regression analysis between color parameters and pigment contents revealed that chlorophyll content exerted a significantly greater influence on the color parameters of *B. × buttiana* ‘Miss Manila’ bracts than betalain or flavonoid content. In summary, while the increased levels of flavonoids and betalains contribute to masking the green color associated with chlorophyll in *B. × buttiana* ‘Miss Manila’ bracts, the reduction in bract chlorophyll content itself is the primary driver underlying the loss of greenness and the increase in redness.

*BgPORA*, a gene involved in chlorophyll biosynthesis, was significantly downregulated during bract development. Conversely, *BgSGR*, which participates in chlorophyll degradation, was significantly upregulated. Concurrently, the contents of betalains and flavonoids increased as the bracts turned red, and *BgDODA*, a gene involved in betalain biosynthesis, was also upregulated. F3H is a key enzyme in the flavonoid biosynthetic pathway [26], regulating the flux towards flavonoids and anthocyanins. CHS is the first committed enzyme in the plant flavonoid pathway [27]. In this experiment, the expression level of *BgCHS* was low at both the bract and bloom stages, suggesting that *BgCHS* is not a key gene for pigment formation in *B. × buttiana* ‘Miss Manila’. *FLS* and *CHIL* are also highly expressed in bracts [28,29]. FLS exerts its respective dominant function in the flavonoid pathway, However, considering the relatively weaker correlation of flavonoid content with color parameters compared to chlorophyll, and the downregulation of the flavonol synthase gene *BgFLS*, the accumulation of flavonoids may play more of an auxiliary coloration or co-pigmentation role rather than being the dominant factor for color display. *CmFLS* serves as an important regulator for the biosynthesis of flavones and flavonols, respectively, and is indicative of flower coloration in *Chrysanthemum morifolium* [30]. *CHIL*-deficient rice mutants were largely depleted of extractable flavones, suggesting their potential involvement in the color changes during *Bougainvillea* bract development [31]. The insights from this study also suggest potential practical applications for the ornamental plant industry. The identified key genes (e.g., *BgDODA*, *BgSGR*) not only enhance our understanding of pigment metabolism but also provide valuable molecular markers for marker-assisted breeding. This opens up avenues for developing new *Bougainvillea* cultivars with novel or intensified bract colors.

## 5. Conclusions

In summary, this study suggests that *B.* × *buttiana* ‘Miss Manila’ bracts, during their early developmental stages (BR1 and BR2), undergo continuous chlorophyll synthesis to sustain energy for subsequent developmental processes. However, in the later developmental stages (BR3 and BR4), chlorophyll degrades, while betalains and flavonoids are synthesized in large quantities. Furthermore, the genes *BgCHIL*, *BgFLS*, *BgDODA*, *BgDFRA*, *BgF3H*, and *BgSGR* may be involved in regulating the color changes in *B. × buttiana* ‘Miss Manila’ bracts.

## Figures and Tables

**Table 1:** RNA template solution system.

Component Name	Volume
Oligo dT Primer (2.5 μM)	1 μL
Template RNA	2 μL
RNase free water	7 μL

**Table 2:** Reverse transcriptional system.

Component Name (200 U/μL)	Volume
the aforementioned reaction solution after denaturation and annealing	10 μL
5× RTase Reaction Buffer II	4 μL
dNTP Mix (10 mM each)	2 μL
Evo M-MLV II RTase	0.5 μL
RNase free water	4.5 μL

**Table 3:** qPCR Reaction Program.

Steps	Temperature	Time	Number of Cycles
Step 1	95 °C	30 s	1
Step 2	95 °C	5 s	40
	60 °C	30 s	
Step 3	Dissociation Stage		

**Table 4:** Primers for qPCR.

Gene Name	Forward Primer (5′ to 3′)	Reverse Primer (5′ to 3′)
*BgDODA*	ACAAAGCGTGGACTTGACCAT	AAGGTTGTAGTGGTGGGTCC
*BgDTX*	TCAACCTTGCTTTGCGCTTG	TACATTTTCGCTGGACCGCT
*BgF3H*	GACACAAGGACTCAGGAGCGATAA	GGGTGGAAGAAGAAGGGAATGGA
*BgCHIL*	CAGTGGATACATTGTTGA	TTCTTCGTCTTCTTCATAA
*BgCHS*	TACTACTTCCGAGTCACT	GTATGTCTTCCGTTAGGT
*BgFLS*	AAGCCAACAATGGAAGTAGA	CTCGGACGAACTCTGATG
*BgSGR*	ATTGCCAAGAACTTACAC	AATGACCACCACTTATGT
*BgDFRA*	CTGCTGTCCTTACTAATAC	TCCAGTGATTCTTGAGAT
*BgPORA*	CTCACAATGCAGGAGTTC	CTCTGAACAAGCCAGTAG
*ACTIN*	TAGACCCTCCTATCCAAACA	TTTTCCAGCCTTCACTTATC

**Table 5:** Color parameters of bracts at different stages of development.

Stages	Lightness L*	Red–Green Component a*	Blue–Yellow Component b*	Chroma C
BR1	52.477 ± 1.829 ^c^	24.78 ± 0.945 ^b^	−4.997 ± 4.096 ^ac^	25.473 ± 1.703 ^b^
BR2	58.123 ± 3.291 ^b^	34.613 ± 3.78 ^b^	−14.2 ± 2.722 ^b^	37.45 ± 4.187 ^b^
BR3	64.773 ± 2.566 ^b^	39.467 ± 4.438 ^b^	−21.247 ± 2.03 ^c^	44.831 ± 4.763 ^b^
BR4	72.98 ± 72.98 ^a^	41.807 ± 1.728 ^a^	−28.843 ± 7.06 ^d^	51.098 ± 2.426 ^a^

Note: Mean (±SD; *n* = 3) (a–d) indicate the significance of *p* < 0.05.

**Table 6:** Pigment content of bracts at different stages of development.

Stages	Chlorophyll a mg/g	Chlorophyll b mg/g	Flavonoid mg/g	Betacyanin mg/g	Betaxanthin mg/g
BR1	1.23 ± 0.10 ^a^	0.97 ± 0.07 ^ac^	1.85 ± 0.01 ^d^	2.93 ± 0.26 ^a^	1.68 ± 0.009 ^a^
BR2	0.75 ± 0.10 ^b^	0.63 ± 0.05 ^b^	2.54 ± 0.09 ^b^	4.45 ± 0.63 ^b^	2.56 ± 0.32 ^c^
BR3	0.47 ± 0.03 ^c^	0.39 ± 0.05 ^c^	3.08 ± 0.04	4.94 ± 0.27 ^b^	2.16 ± 0.19 ^b^
BR4	0.27 ± 0.03 ^d^	0.34 ± 0.04 ^d^	2.04 ± 0.003 ^c^	4.69 ± 0.06 ^b^	2.27 ± 0.05 ^bc^

Note: One-way analysis of variance (ANOVA). Mean (±SD; *n* = 3) (a–d) indicate the significance of *p* < 0.05.

**Table 7:** Multiple linear regression analysis.

Color Parameters	Multiple Linear Regression	R^2^
Chroma C	y = −27.80681 − 35.32951 × chlorophyll a content + 78.26294 × chlorophyll b content + 22.6553 × flavonoid content + 0.47252 × betacyanin content − 6.04786 × betaxanthin content	0.925
L*	y = 32.19816 − 27.053 × chlorophyll a content + 55.10084 × chlorophyll b content + 5.82794 × flavonoid content + 4.08936 × betacyanin content − 8.20242 × betaxanthin content	0.997
a*	y = 15.43201 − 27.15873 × chlorophyll a content + 46.64347 × chlorophyll b content + 10.36714 × flavonoid content − 0.83358 × betacyanin content − 5.91821 × betaxanthin content	0.851
b*	y = 103.13033 + 26.2152 × chlorophyll a content − 88.89036 × chlorophyll b content − 31.11977 × flavonoid content − 2.15693 × betacyanin content − 0.89268 × betaxanthin content	0.946

## Data Availability

All data generated/analyzed during this study are included in this article and its Supplementary Files.

## Supplementary Materials

The following supporting information can be downloaded at: https://www.mdpi.com/article/10.3390/biology14111607/s1, Figure S1: Signal peptide prediction of BgFLS ‘Psignal’ or ‘Signal’; Figure S2: Transmembrane structure prediction of BgFLS; Figure S3: Tertiary structure prediction of BgFLS; Figure S4: Signal peptide prediction of BgCHIL; Figure S5: Transmembrane structure prediction of BgCHIL; Figure S6: Tertiary structure prediction of BgCHIL.

## Abbreviations

The following abbreviations are used in this manuscript:

L*	Lightness
a*	Red–green component
b*	Blue–yellow component
C	Chroma
DODA	4,5-DOPA dioxygenase gene
RT-qPCR	Real-time quantitative PCR
SGR	STAY-GREEN
POR	protochlorophyllide oxidoreductase
DFR	dihydroflavonol 4-reductase
FLS	flavonol synthase/flavanone 3-hydroxylase
CHIL	Chalcone-flavanone isomerase family protein
