# Supplementary material for: Analysis of Pigmentation Changes in Bracts of Bougainvillea × buttiana ‘Miss Manila’ During Different Developmental Periods
Source: Biology (Basel). 2025 Nov 17;14(11):1607. doi: 10.3390/biology14111607 (PMC12650391; doi:10.3390/biology14111607)
Supplement: Supplementary file 1 [file biology-14-01607-s001.zip › biology-3903594-supplementary.pdf]

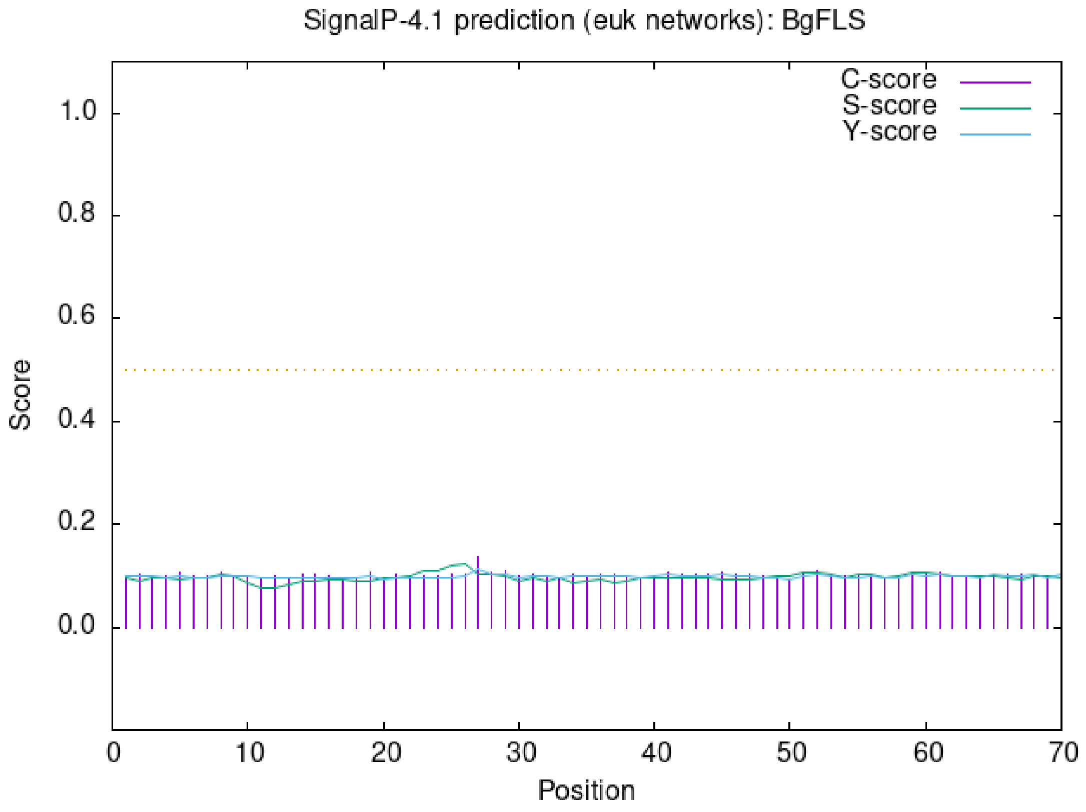

Fig. S1 Signal peptide prediction of BgFLS 'Psignal' or 'Signal'

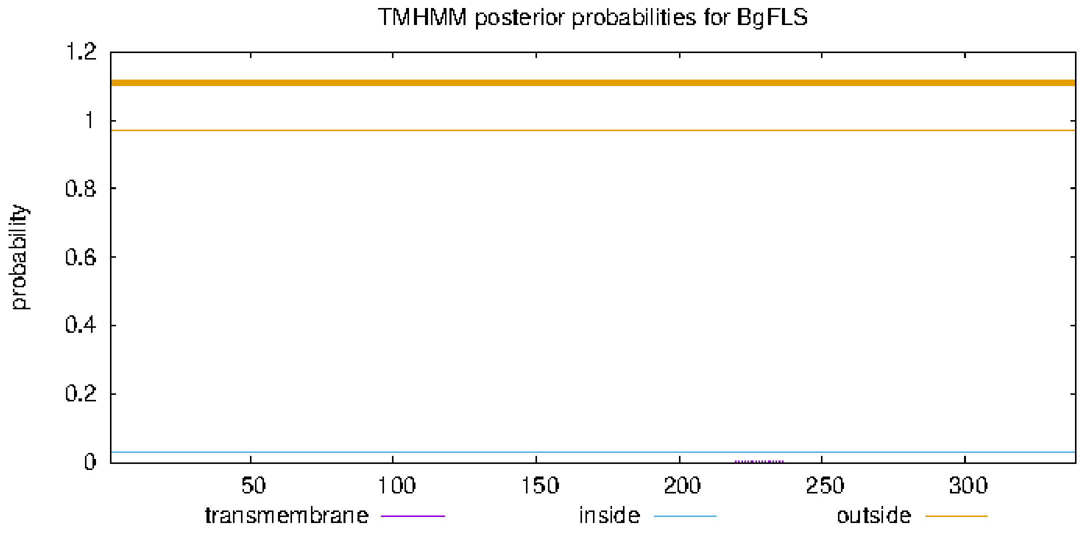

Fig. S2 Transmembrane structure prediction of BgFLS

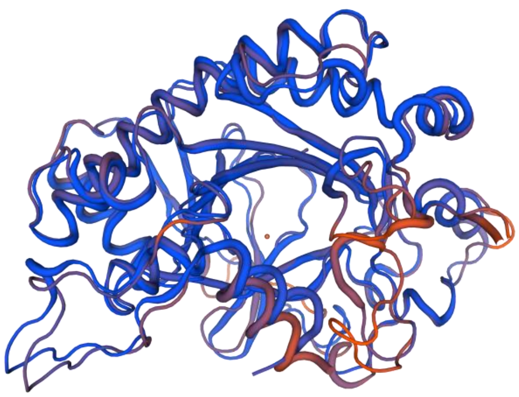

Fig. S3 Tertiary structure prediction of BgFLS

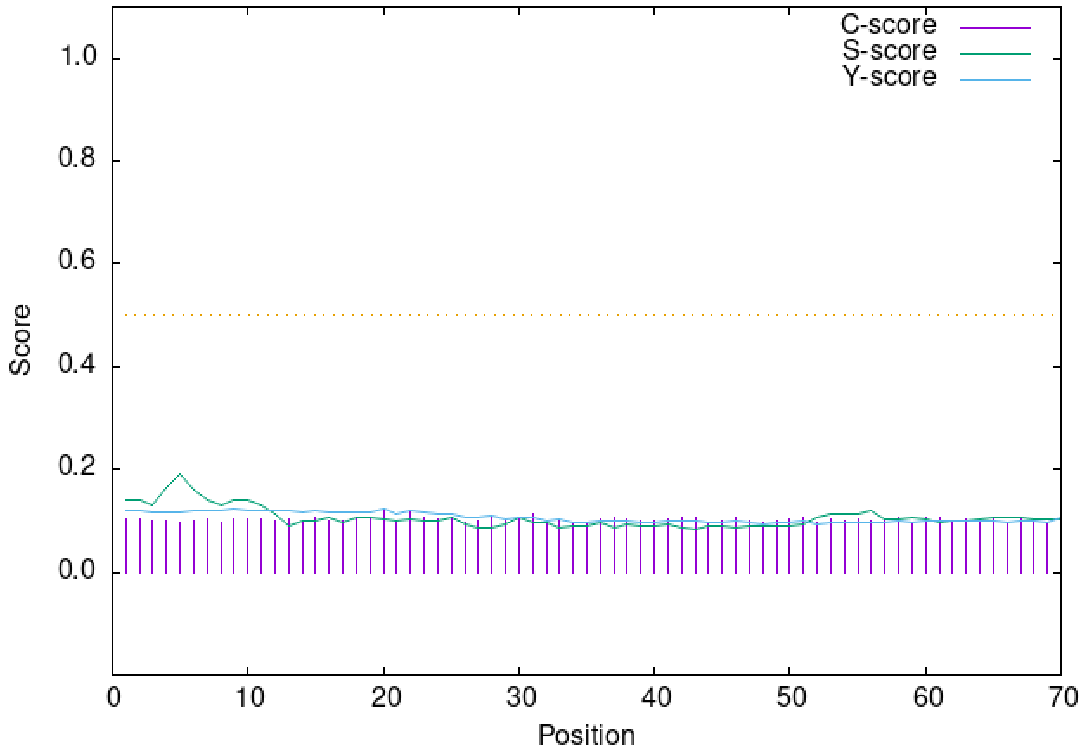

Fig. S4 Signal peptide prediction of BgCHIL

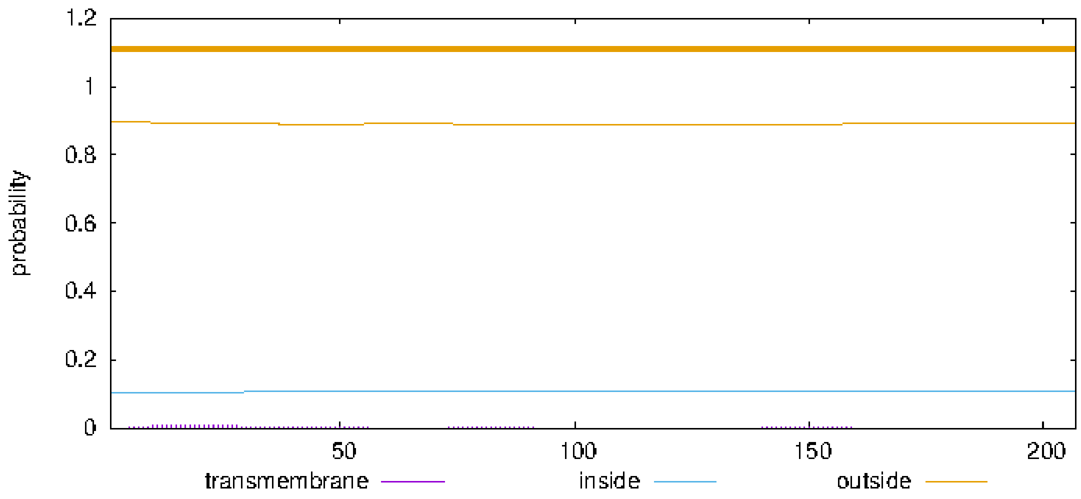

Fig. S5 Transmembrane structure prediction of BgCHIL

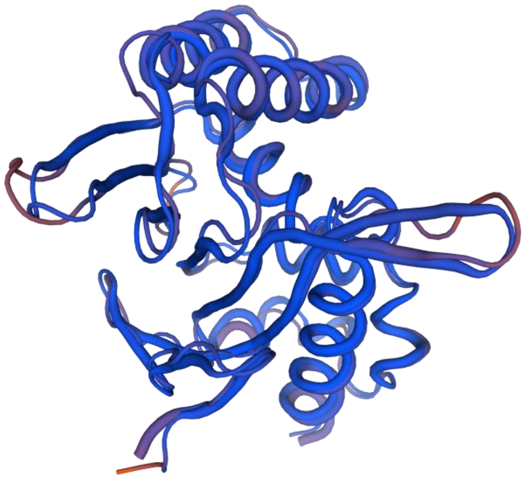

Fig. S6 Tertiary structure prediction of BgCHIL
